# Exploring the Causal Effects of Physical Activity, Sedentary Behaviour, and Diet on Atrial Fibrillation and Heart Failure: A Multivariable Mendelian Randomisation Analysis

**DOI:** 10.3390/nu16234055

**Published:** 2024-11-26

**Authors:** Yunong Zhang, Ye Tao, Hyunsoo Choi, Haonan Qian

**Affiliations:** 1 Department of Physical Education, Sejong University, Seoul 05006, Republic of Korea; yunong03@sju.ac.kr (Y.Z.); ty231004@sju.ac.kr (Y.T.); 2 Department of Physical Education, Hanyang University, Seoul 04763, Republic of Korea; ypd98014@hanyang.ac.kr

**Keywords:** multivariate Mendelian randomization, physical activity, sedentary behaviour, diet, atrial fibrillation, heart failure, causal effects

## Abstract

Aims: This study aimed to investigate the causal effects of physical activity, sedentary behaviour, and diet on atrial fibrillation (AF) and heart failure (HF) using multivariate Mendelian randomization (MR) analysis and genetic variants as instrumental variables. Methods: The study employed multivariate MR analysis with physical activity, sedentary behaviour, and diet as exposures and AF and HF as outcomes. Data were obtained from the UK Biobank (over 500,000 participants) and the FinnGen project (218,792 participants of European ancestry). Genetic variants associated with physical activity, diet, and sedentary behaviour were used as instrumental variables. The main analysis methods included the inverse variance weighted (IVW) method, MR-Egger, and weighted median methods. Heterogeneity was assessed using Cochran’s Q test. Results: The analyses generally did not demonstrate significant causal relationships between physical activity or sedentary behaviour and AF. Diet showed a potential protective effect on AF in some analyses but was not consistently significant across methods. For HF, physical activity and sedentary behaviour did not show significant causal relationships. Diet showed a significant protective effect against HF in the IVW method but was not consistent across all methods. Conclusions: This study suggests that while there may be some protective effects of these lifestyle factors on cardiovascular disease, most analyses did not show significant causality, and results were inconsistent. Further research is needed to validate these findings.

## 1. Introduction

Atrial fibrillation (AF) and heart failure (HF) are two major cardiovascular diseases affecting millions of people worldwide. AF is a common arrhythmia affecting approximately 33 million people worldwide [1], with its incidence and prevalence increasing significantly with age. According to epidemiological data, the prevalence of AF ranges from 4 to 5% in people over 65 years of age to even more than 10% in people over 80 years of age [2,3]. In recent years, with the ageing of the global population and changes in lifestyle, the incidence of AF has been on the rise year after year [4].

Heart failure, on the other hand, is a clinical syndrome characterised by the inability of the heart to effectively pump enough blood to meet the body’s needs. It is estimated that about 26 million people worldwide suffer from HF [5], with a prevalence of about 1–2% in developed countries and up to 10% or more in people over 65 years of age. The prevalence of HF increases significantly with age and is slightly higher in men than in women [6]. Similarly to AF, the prevalence of HF is increasing each year as the population ages and the incidence of cardiovascular disease increases.

Both diseases not only have a high global prevalence but also impose a significant economic burden on public health and healthcare systems. Patients with AF and HF usually require frequent medical interventions and long-term medication, which not only increases healthcare costs but also has a profound impact on patients’ quality of life [7].

AF and heart failure are not only standalone health problems but can lead to a variety of serious complications that can further aggravate a patient’s condition and life. AF is associated with a number of serious complications, such as stroke, heart failure [8], and cognitive dysfunction. Patients with AF have a five times higher risk of stroke and a significantly increased risk of thrombosis due to the abnormal blood flow caused by AF [9]. Once dislodged, these clots can lead to stroke and permanent neurological damage. In addition, AF causes structural and functional changes in the atria, which, in turn, increases the risk of heart failure [10].

Heart failure itself is a progressive disease, with complications including acute decompensation, cardiogenic shock, renal insufficiency, pulmonary oedema, etc. [11]. The poor prognosis of patients with HF, with a five-year survival rate of less than 50%, is due to the fact that heart failure is often accompanied by other cardiovascular diseases such as coronary artery disease, hypertension, and diabetes mellitus [12]. In addition, patients with HF often experience symptoms such as fatigue, dyspnoea and oedema, which severely affect their daily life and ability to work.

The complications of these two diseases not only increase the morbidity, mortality, and disability rates of patients but also impose heavy economic and psychological burdens on families and society. Therefore, the prevention and control of AF and HF, as well as their complications [13,14,15], have become an important task for global public health.

Given the high prevalence and severe complications of AF and HF, it is particularly important to explore effective prevention and treatment strategies [16,17]. In recent years, an increasing number of studies have shown that lifestyle factors, such as physical activity, sedentary behaviour and dietary habits, have a significant impact on cardiovascular health [18].

Physical activity is widely recognised as an effective measure for the prevention of cardiovascular disease. Moderate aerobic exercise improves cardiovascular function, lowers blood pressure, improves the lipid profile, and reduces inflammatory response, thereby reducing the risk of AF and HF [18]. However, further research is needed on the specific causal effects of physical activity on AF and HF, especially the effects of different types and intensities of exercise on these diseases [19].

Sedentary behaviour, especially prolonged sitting, is strongly associated with the development of several cardiovascular diseases. Sedentary behaviour not only increases the risk of obesity and metabolic syndrome [20,21] but may also lead to the deterioration of cardiovascular function. Studies have shown that reducing sedentary time and increasing daily activity can significantly improve cardiovascular health. However, the direct causal effect of sedentary behaviour on AF and HF remains unclear and needs to be further explored [22].

As an important protective factor for cardiovascular health, activity has been widely studied to prove its preventive effect on atrial fibrillation and heart failure. However, the rise in sedentary behaviours (such as watching TV for long periods of time, using computers, etc.) poses new threats to cardiovascular health [23]. Existing research shows that sedentary behaviour is closely related to the risk of metabolic syndrome, type 2 diabetes, and cardiovascular disease, but its specific mechanisms have not been fully elucidated [24]. As for dietary factors, the impact of different dietary structures on atrial fibrillation and heart failure also shows a complex trend. For example, high-fat and high-salt diets may increase the risk of disease, while foods rich in fibre may have a protective effect [25]. The current body of literature presents conflicting findings regarding the impact of activity, sedentary behaviour, and diet on AF and HF. One contributing factor to this inconsistency is the lack of harmonisation in the definition and measurement standards for sedentary behaviour, which complicates the comparison of results across different studies [26]. Additionally, while Mendelian randomisation (MR) analysis offers a robust approach to causal inference, its outcomes can be influenced by the complexity of genetic variation, sample size, and population differences, potentially introducing uncertainty [27]. Furthermore, the interplay between activity, sedentary behaviour, and diet, as well as their combined effects with other environmental factors, have not been fully elucidated.

To address these gaps, our study employs multivariate MR analysis, a robust method that minimises the influence of confounders and reverse causality in observational studies, thereby providing more reliable causal inferences [28,29,30]. By utilising genetic variants as instrumental variables, we aim to explore the causal effects of physical activity, sedentary behaviour, and diet on AF and HF [31]. This approach not only seeks to uncover the specific mechanisms by which these lifestyle factors contribute to disease development but also aims to furnish a scientific foundation for the formulation of personalised prevention and treatment strategies. Our research, thus, fills an important gap in the current understanding of lifestyle-related disease mechanisms and offers a novel perspective on the development of targeted interventions.

## 2. Methods

### 2.1. Study Design

This study employed a rigorous multivariate Mendelian randomisation analysis framework to delve into the causal mechanisms underlying the effects of physical activity, sedentary behaviour, and dietary patterns on atrial fibrillation and heart failure. By incorporating multiple exposure factors, including but not limited to physical activity levels, sedentary habits, and dietary components, this study aimed to provide a comprehensive assessment of their individual and combined impacts on these cardiovascular outcomes. AF and heart failure were then used as outcome variables. There are three key assumptions that need to be met in order to conduct a Mendelian randomisation study: first, the selected single nucleotide polymorphisms (SNPs) should be significantly associated with exposure factors (e.g., physical activity, diet, and nutrition); second, the SNPs should be independent of potential confounders; and, third, the SNPs can only be associated with the outcome variables (e.g., physical activity and heart failure) through exposure factors. This study used pooled data from published studies that had received informed consent and ethical approval from participants.

### 2.2. Data Resources

The UK Biobank (UKB) and FinnGen are large prospective cohort studies that have successfully recruited more than 500,000 men and women aged between 40 and 69 years, focusing on their long-term health status. The FinnGen study included 218,792 participants of European origin (68,782 cases and 150,010 controls). These data were used to assess the effects of physical activity, sedentary behaviour, and diet on AF and heart failure. ukb-b-8764(SNPs:9,851,867)/ebi-a-GCST90061428(SNPs:8,489,912)/UKB-E-1538_p1_AFR(SNPs:15,533,042)/ebi-a-GCST006414(SNPs:33,519,037)/ebi-a-GCST90018806(SNPs:24,178,220).

### 2.3. Selection of Genetic Instrumental Variables

Genetic variants associated with physical activity, diet, and nutrition were selected as instrumental variables (IVs) if they reached a genome-wide significance level (*p* < 5 × 10^−8^). To avoid bias due to linkage disequilibrium, independent SNPs (defined as r^2^ < 0.001 and a clustering window ≥ 10,000 kb) were selected. To correct the orientation of the alleles, the SNPs were coordinated. The formula F = R^2^(N − K − 1)/[K(1 − R^2^)] was used to screen the F-statistic of SNPs that were highly correlated with exposure factors [32]. In this formula, R^2^ is the cumulative explained variance in the selected SNP in the exposure factor, N is the sample size of the exposure database, and K is the number of SNPs ultimately analysed. The F-statistic for each instrument-exposure effect ranged from 26.604 to 26.992, indicating a low likelihood of weak instrumental bias.

### 2.4. Statistical Analysis Methods

In the magnetic resonance imaging analysis of this study, inverse variance weighting (IVW) was used as the primary analysis method. Heterogeneity among the estimates of genetic variants was assessed using Cochran’s Q test. If the *p*-value of Cochran’s Q test was less than 0.05, a random effects model was used to analyse the final results of the MR analysis; otherwise, a fixed effects model was applied. As a supplementary analysis to IVW, weighted median, maximum likelihood estimation, MR-Egger regression, and penalised weighted median methods were employed. Additionally, we applied a recently proposed MR method called the robust adjusted profile score (RAPS), which corrects for polygenic bias using robust adjusted profile scores to reduce the bias caused by this. Finally, the MR-PRESSO (MR Pleiotropy RESidual Sum and Outlier) was used to validate the results of the IVW model, testing and calibrating for horizontal pleiotropy outliers.

### 2.5. Sensitivity Analysis

To identify potential pleiotropic effects, the MR-Egger test was conducted. If the *p*-value of the MR-Egger intercept was greater than 0.05, it indicated the absence of horizontal pleiotropy. Exclusion sensitivity analysis was performed by sequentially excluding SNPs to evaluate the stability of the results. Furthermore, funnel plots and forest plots were generated to directly explore the presence of pleiotropy effects.

## 3. Results

**Figure 1:**
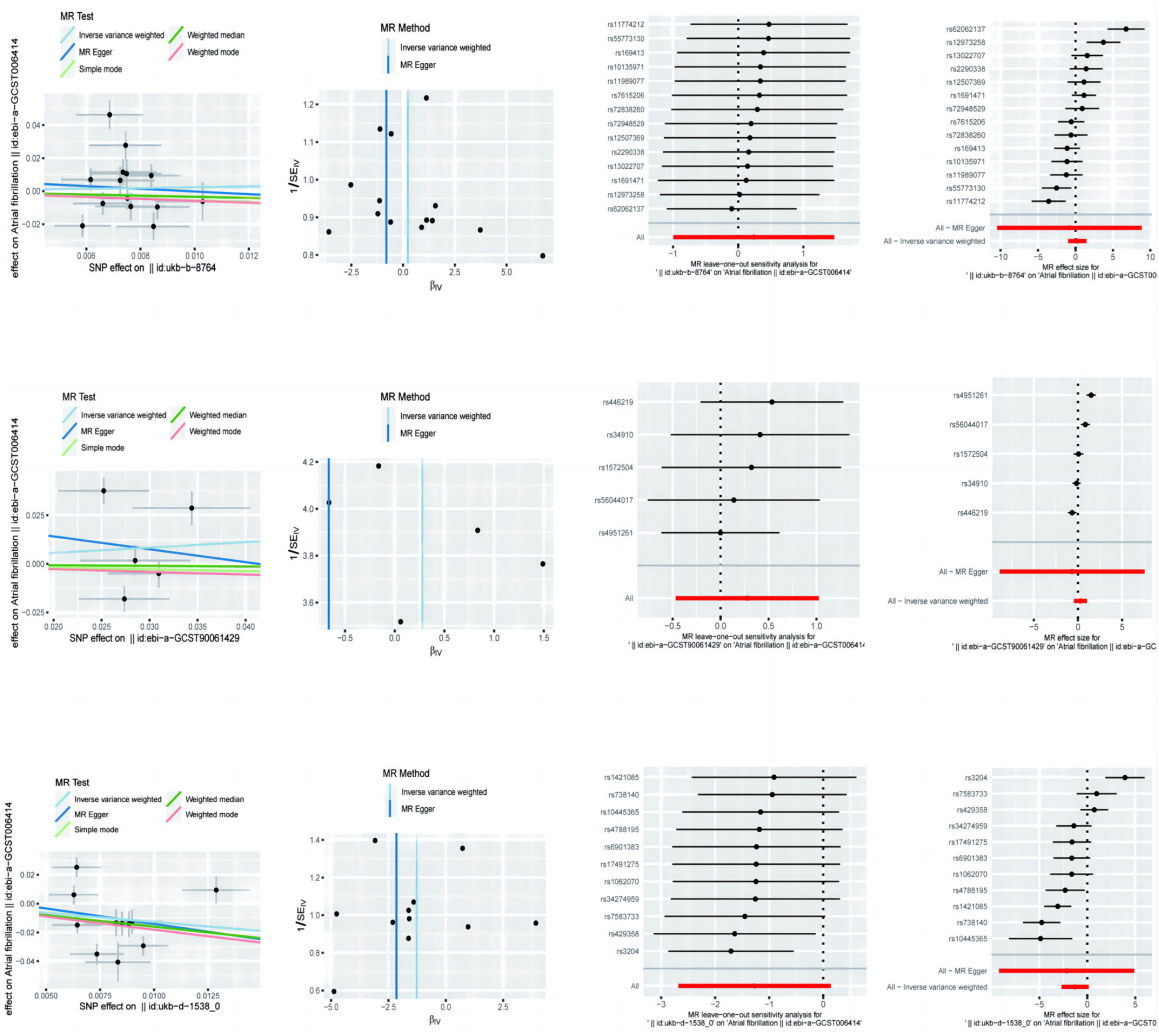
Mendelian randomisation of exercise, diet, and nutrition in atrial fibrillation.

In this paper, we analysed the results of the assessment of the causal effect of different lifestyle factors, including physical activity, sedentary behaviour and diet, on AF. Three different methods of MR analysis are presented in the table (Table 1, Figure 1), i.e., MR-Egger, weighted median, and inverse variance weighted, and the odds ratio (OR) and 95% confidence interval (CI), upper and lower bounds, for each method are given. (The odds ratio (OR), 95% confidence interval (CI) upper and lower limits, *p*-value, and Beta value were given for each method).

For the effect of physical activity on AF, MR-Egger’s analysis showed an OR of 0.4451, 95% CI of 2.85 × 10^−5^ to 6954.7211, a *p*-value of 0.8723, and a Beta value of −0.8093, which suggests that there is no significant causal relationship between physical activity and AF. The results of the weighted median method showed an OR of 0.7115 with a 95% CI of 0.2823 to 1.7930, a *p*-value of 0.4704, and a Beta value of −0.3404, which also failed to show a significant causal relationship. Analysis of the inverse variance weighting method showed an OR of 1.2707, 95% CI of 0.3696 to 4.3686, a *p*-value of 0.7038, and a Beta value of 0.2396, again failing to demonstrate the significant effect of physical activity on AF.

To analyse the effect of sedentary behaviour on AF, MR-Egger’s method showed an OR of 0.5151, 95% CI of 0.0001391 to 1907.0899, a *p*-value of 0.8843, and a Beta value of −0.6634, which indicated no significant causal relationship between sedentary behaviour and AF. The results of the weighted median method showed an OR of 0.9664 with a 95% CI of 0.6571 to 1.4214, a *p*-value of 0.8623, and a Beta value of −0.0341, which again failed to show a significant causal relationship. Analysis of the inverse variance weighting method showed an OR of 1.3217, 95% CI of 0.6265 to 2.7886, a *p*-value of 0.4640, and a Beta value of 0.2789, failing to demonstrate a significant effect of sedentary behaviour on AF.

The effect of diet on AF was analysed. MR-Egger’s analysis showed an OR of 0.1163, 95% CI of 0.0001009 to 134.0898, a *p*-value of 0.5645, and a Beta value of −2.1516, suggesting that there was no significant causal relationship between diet and AF. The results of the weighted median method showed an OR of 0.2012, 95% CI of 0.0718 to 0.5640, a *p*-value of 0.0023, and a Beta value of −1.6034, indicating a significant protective effect of diet on AF. The inverse variance weighted method analysis showed an OR of 0.2806, 95% CI of 0.0684 to 1.1514, a *p*-value of 0.0777, and a Beta value of −1.2710, suggesting that the diet may have a protective effect on AF, but the results did not reach statistical significance.

To assess the potential causal relationship between these exposures and AF and heart failure, we performed sensitivity analyses, including tests of heterogeneity and tests of pleiotropy. Table 2 shows the results of sensitivity analyses by different methods. The results of the heterogeneity test (heterogeneity test) and the pleiotropy test (pleiotropy test) were as follows:

Physical Activity: for the heterogeneity test using the MR-Egger method, the Q-value was 66.0877, the degree of freedom (*df*) was 12, and the *p*-value was 1.71 × 10^−9^; using the inverse variance weighted (IVW) method, the Q-value was 66.3418, the degree of freedom was 13, and a *p*-value of 3.76 × 10^−9^ was used. Multiple validity test: the MR-Egger intercept term (intercept) was 0.0079, the standard error (SE) was 0.0368, and the *p*-value was 0.8335.

Sedentary Behaviour (SB): for the heterogeneity test using the MR-Egger method, the Q-value was 43.1053, with three degrees of freedom and a *p*-value of 2.34 × 10^−9^; using the IVW method, the Q-value was 43.8392, with four degrees of freedom and a *p*-value of 6.93 × 10^−9^. Tests of multivalence: the MR-Egger intercept term was 0.0274, the standard error was 0.1212, and the *p*-value was 0.8357.

Diet (Diet): For the heterogeneity test, using the MR-Egger method, the Q-value was 60.8629, with nine degrees of freedom and a *p*-value of 9.14 × 10^−10^; using the IVW method, the Q-value was 61.2870, with ten degrees of freedom and a *p*-value of 2.07 × 10^−9^. Test of multiple validity: the MR-Egger intercept term was 0.0074, the standard error was 0.0295, and the *p*-value was 0.8079.

The results of the heterogeneity test in the table show that all three exposure factors have high Q-values and very small *p*-values, indicating significant heterogeneity, which suggests that there may be unexplained heterogeneity or that some assumptions of the model are not valid. The *p*-value of the intercept term of the MR-Egger method in the test of multicollinearity was greater than 0.05, indicating no significant multicollinearity.

**Figure 2:**
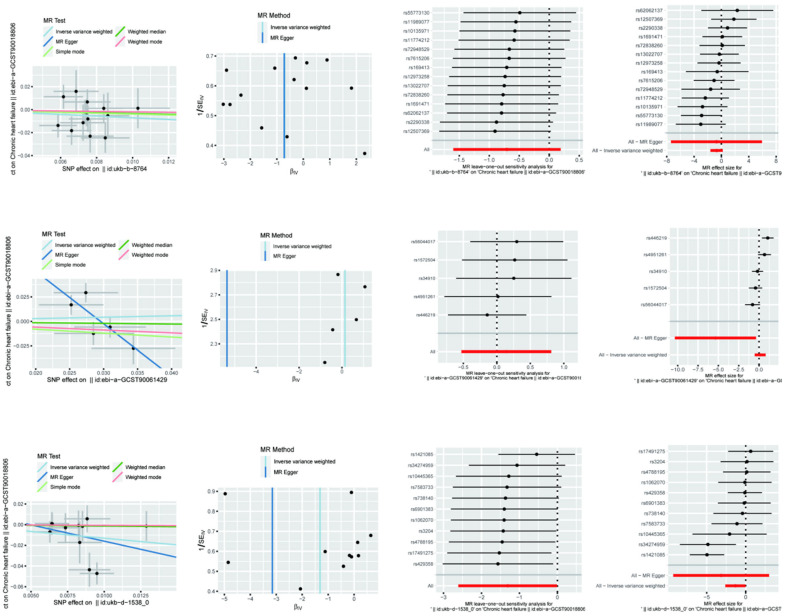
Mendelian randomisation of exercise, diet, and nutrition on heart failure.

For the effect of physical activity (physical activity) on heart failure (Table 3 and Figure 2), the OR for MR-Egger analysis was 0.486590469 with a 95% confidence interval of 0.000624439 to 379.1725789, a *p*-value of 0.835632315, and a Beta value of −0.720332436. The weighted median method had an OR of 0.712369619 with a 95% confidence interval of 0.203855562 to 2.489362905, a *p*-value of 0.595213274, and a Beta value of −0.339158374. The inverse variance weighted method had an OR of 0.4941075 with a 95% confidence interval of 0.20185207 to 1.209510614, a *p*-value of 0.122699954, and a Beta value of −0.705002175. The above results show that although the effect estimates of all the analytical methods were less than one, suggesting that physical activity may have a protective effect against heart failure, their statistical significance was not strong.

For the effect of sedentary behaviour (sedentary behaviour) on heart failure, the OR value of the MR-Egger analysis was 0.004708406 with a 95% confidence interval of 3.22 × 10^−5^ to 0.689278146, a *p*-value of 0.125812618, and a Beta value of −5.35840585. The weighted median method had an OR of 0.934784168 with a 95% confidence interval of 0.553544478 to 1.578593005, a *p*-value of 0.8008341, and a Beta value of −0.067439613. The inverse variance weighted method had an OR of 1.152275643 with a 95% confidence interval of 0.589378864 to 2.252777013 with a *p*-value of 0.678598673 and a Beta value of 0.141738807. Although MR-Egger’s analysis showed that sedentary behaviour may have a significant protective effect against heart failure, this result was not consistently supported by the weighted median method and the inverse variance weighted method and, therefore, needs to be interpreted with caution.

For the effect of diet (diet) on heart failure, the MR-Egger analysis had an OR of 0.042792355 with a 95% confidence interval of 9.05 × 10^−5^ to 20.22914228, a *p*-value of 0.342082949, and a Beta value of −3.151395824. The weighted median method had an OR of 0.877123612 with a 95% confidence interval of 0.253391575 to 3.036193412, a *p*-value of 0.836050089, and a Beta value of −0.131107348. The inverse variance weighted method had an OR of 0.269903463 with a 95% confidence interval of 0.073593038 to 0.989874611, a *p*-value of 0.048228497, and a Beta value of −1.309690927. The results of the inverse variance weighted method showed that diet had a significant protective effect against heart failure with a statistically significant OR value significantly less than one and a *p*-value less than 0.05, but the results of other methods of analysis were not significantly consistent.

In Table 4 of this paper, we performed sensitivity analyses and multivariate Mendelian randomisation analyses on the causal effects of physical activity, sedentary behaviour, and diet on heart failure. This table details the results of the tests of heterogeneity and tests of multivariate validity derived from different statistical methods aimed at assessing the reliability and robustness of the potential causal effects of these lifestyle factors on heart failure.

For physical activity, we first conducted heterogeneity tests using the MR-Egger and inverse variance weighted (IVW) methods, respectively. The Q-statistic for the MR-Egger method was 11.33521276, with a degree of freedom of 12 and a *p*-value of 0.500430229, which indicated that the data did not have significant heterogeneity. Similarly, the IVW method had a Q-statistic of 11.3352335, a degree of freedom of 13, and a *p*-value of 0.582756241, which also indicated that there was no significant heterogeneity in the data. In addition, the test of multivariate validity by the MR-Egger method yielded an intercept of 0.000114603, a standard error of 0.025164734, and a *p*-value of 0.996441205, which showed that there was no significant multivariate validity for the physical activity variable.

The results of the heterogeneity test for sedentary behaviour were different. The MR-Egger method had a Q-statistic of 5.921246215 with a degree of freedom of three and a *p*-value of 0.115505269, which indicated that there was no significant heterogeneity in the data. However, the IVW method had a Q-statistic of 15.23412951 with a degree of freedom of four and a *p*-value of 0.004239456, which indicated significant heterogeneity. The results of the multivariate test showed that the MR-Egger method obtained an intercept of 0.158301685, a standard error of 0.072876816, and a *p*-value of 0.118213505, indicating that there was also no significant multivariate validity for the sedentary behaviour variable.

For diet, both the MR-Egger and IVW methods showed the presence of significant heterogeneity. The MR-Egger method had a Q-statistic of 19.287684 with a degree of freedom of nine and a *p*-value of 0.022854908, while the IVW method had a Q-statistic of 20.0607247 with a degree of freedom of ten and a *p*-value of 0.028683538. Regarding the tests for multivalence, the results showed that the MR-Egger method yielded an intercept of 0.015535499, a standard error of 0.025866806, and a *p*-value of 0.562926009, indicating that there was no significant multivalence for the dietary variables.

Taken together, the results of these sensitivity analyses suggest that the causal effects of physical activity, sedentary behaviour, and diet on heart failure were heterogeneous in some cases but did not show significant pleiotropy overall.

## 4. Discussion

Atrial fibrillation (AF) is the most common persistent arrhythmia worldwide and is characterised by rapid and irregular electrical activity in the atria, resulting in the inability of the atria to efficiently pump blood into the ventricles [33]. This abnormal electrical activity causes the uncontrolled contraction of the atrial muscle fibres, which ultimately affects the overall pumping function of the heart. According to epidemiological studies [34,35,36,37], and all-cause mortality, in particular due to blood stagnation and vortex formation in the atria, the likelihood of thrombus formation is greatly increased, and once these clots enter the circulation, they may lead to severe ischaemic stroke [38].

The symptoms of AF are diverse; some patients may be completely asymptomatic, while others may experience uncomfortable symptoms such as palpitations, chest pain, breathlessness, fatigue, and fainting. This symptomatic diversity and uncertainty make diagnosis more difficult [39], resulting in many patients failing to seek medical attention in the early stages of symptoms and missing out on optimal treatment. Although current treatment options include pharmacological therapy, electrical cardioversion, catheter ablation, and left ear blockade, the outcome and prognosis of treatment vary with individual patients [40,41,42,43]. Electrical cardioversion and catheter ablation, as non-pharmacological treatments, have shown better results in restoring normal rhythm, but their high costs and procedural risks also limit their widespread use [43]. Therefore, in-depth research and exploration of the etiology of AF and its relationship with lifestyle factors are essential for developing more effective prevention and treatment strategies [44].

Heart failure (HF) is a syndrome caused by abnormalities in the structure and function of the heart, manifested by the heart’s inability to pump blood with the efficiency required to meet the body’s metabolic demands. According to its causes and manifestations, heart failure can be divided into two categories: systolic heart failure and diastolic heart failure [45,46,47]. In either type of heart failure, the patient’s quality of life is severely affected, and the incidence of heart failure is high, as is the mortality rate. Globally, the prevalence and incidence of heart failure are on the rise [48,49,50], especially in the context of ageing societies and the high prevalence of chronic diseases. According to statistics, the prevalence of heart failure in developed countries is around 1 to 2 per cent, while in the elderly population aged 75 years and above, it can be as high as 10 per cent or more [51].

Clinical symptoms of heart failure include dyspnoea, fatigue, weakness, oedema, nocturnal paroxysmal dyspnoea, etc. These symptoms will worsen with the progress of the disease and ultimately lead to a significant reduction in the patient’s daily life and working ability [52]. The causes of heart failure are complex and may be caused by a variety of cardiac and systemic diseases, such as coronary artery disease, hypertension, diabetes mellitus, cardiomyopathy, and heart valve disease [53,54,55]. In addition, lifestyle factors such as smoking, alcohol consumption, poor dietary habits, and lack of physical activity are also considered important risk factors for heart failure [56]. Despite significant advances in the treatment of heart failure in modern medicine, such as pharmacological therapy, cardiac resynchronisation therapy, implantable cardioverter–defibrillators, and heart transplantation, a significant proportion of patients still have a poor prognosis and limited therapeutic benefits [57]. The prevention and management of heart failure require comprehensive multidisciplinary interventions, including the optimisation of pharmacological therapy and the improvement of lifestyle and psychological support [58,59,60,61].

In this study, we explored the causal effects of physical activity, sedentary behaviour, and diet on atrial fibrillation (AF) and heart failure (HF) by multivariate Mendelian randomisation (MR) analysis. Although multiple analytical methods yielded not entirely consistent results, our study provides some important insights.

For AF, we found differential causal effects of physical activity, sedentary behaviour, and diet. The associations between physical activity and sedentary behaviour and AF did not show significance across the different MR analysis methods. Specifically, the MR-Egger method showed an OR of 0.4451 (95% CI: 2.85 × 10^−5^ to 6954.7211, *p* = 0.8723) for physical activity and 0.5151 (95% CI: 0.0001391 to 1907.0899, *p* = 0.8843) for sedentary behaviour. In contrast, diet showed a protective effect in the weighted median and inverse variance weighted methods (OR = 0.2012, 95% CI: 0.0718 to 0.5640, *p* = 0.0023; OR = 0.2806, 95% CI: 0.0684 to 1.1514, *p* = 0.0777).

For HF, the results showed a possible protective effect of physical activity, but it was not statistically significant. The OR was 0.4866 (95% CI: 0.0006244 to 379.1726, *p* = 0.8356) for the MR-Egger method and 0.7124 (95% CI: 0.2039 to 2.4894, *p* = 0.5952), while the inverse variance weighted method had an OR of 0.4941 (95% CI: 0.2019 to 1.2095, *p* = 0.1227). Sedentary behaviour had inconsistent results across methods, showing a non-significant causal relationship with HF, whereas diet showed a significant protective effect in the inverse variance weighting method (OR = 0.2699, 95% CI: 0.0736 to 0.9899, *p* = 0.0482).

The benefits of physical activity in combating cardiovascular disease are widely recognised, but our study failed to demonstrate a significant causal effect on AF and HF. Possible reasons for this include the selection of genetic instrumental variables and sample size limitations. Although the F-statistic showed adequate strength for the instrumental variables, genetic instrumental variables alone may not be sufficient to capture all relevant biological pathways due to the multifactorial etiological nature of AF and HF. In addition, the magnitude of physical activity in the study may not be sufficient to induce significant physiological changes, or the protective effect of physical activity on AF and HF may require a longer follow-up period to become apparent.

Sedentary behaviour is considered to be one of the risk factors for cardiovascular disease. However, our findings did not show a significant causal effect on AF and HF. This may be related to the definition and measurement of sedentary behaviour. The instrumental variables we used may not be sufficient to accurately reflect the complexity and diversity of sedentary behaviour. In addition, the interaction of sedentary behaviour with other lifestyle factors (e.g., diet, weight, exercise) may influence its impact on cardiovascular health.

The protective effect of diet on AF and HF was partially validated in our study. In particular, the inverse variance weighting method showed the significant protective effect of diet on HF. Dietary habits, especially the Mediterranean diet and diets rich in dietary fibre, are thought to contribute to a reduction in CVD risk. Our results support this view, emphasising the importance of a healthy diet in the prevention of CVD. However, the inconsistency of the results between the different analytical methods also suggests that further studies are needed to confirm the specific mechanism of action of diets.

The main strength of this study is the use of multivariate Mendelian randomisation analysis, which is a powerful tool to reduce the effects of potential confounders and reverse causality in observational studies. By using large-scale data from UK Biobank and FinnGen, we were able to conduct analyses with high statistical power. However, there are some limitations to this study.

Although we selected SNPs that reached genome-wide significance levels as instrumental variables, these may not be sufficient to fully capture the complexity of physical activity, sedentary behaviour, and diet. Second, despite the large sample size, a single genetic instrumental variable may not adequately represent the diversity of exposure factors due to the complexity of the pathogenesis of AF and HF. In addition, the present study was primarily based on a European population, and the generalisability of the results may be limited and not necessarily applicable to other ethnicities and populations.

Future studies should further explore the effects of physical activity, sedentary behaviour, and diet on CVD, especially with finer instrumental variables and larger cohort data. In addition, attention should be paid to differences across ethnicities and populations to increase the generalisability of our findings. Specifically, research on dietary patterns should be strengthened to combine nutritional and metabolomic data to reveal the protective mechanisms of diet on cardiovascular health.

## 5. Conclusions

In summary, this study explored the causal effects of physical activity, sedentary behaviour, and diet on AF and HF using multivariate Mendelian randomisation analysis. Although the results showed that physical activity and sedentary behaviour were not significantly causally related to AF and HF, the protective effect of diet on HF was partially verified. Future studies should continue to deepen exploration in this area, with a view to providing a more scientific basis for the prevention and treatment of cardiovascular diseases.

## Figures and Tables

**Table 1:** Mendelian randomisation of physical activity, diet and sedentary behaviour on atrial fibrillation.

		Atrial Fibrillation				
		or	or_lci95	or_uci95	*p*-Value	Beta
Physical Activity	MR-Egger	0.445	2.85 × 10^−5^	6954.721064	0.872	−0.809
	Weighted median	0.711	0.282	1.793	0.470	−0.340
	Inverse variance weighted	1.271	0.370	4.369	0.704	0.240
Sedentary Behaviour	MR-Egger	0.515	0.0001	1907.090	0.884	−0.663
	Weighted median	0.966	0.657	1.421	0.862	−0.034
	Inverse variance weighted	1.322	0.626	2.789	0.464	0.279
Diet	MR-Egger	0.116	0.0001	134.090	0.564	−2.152
	Weighted median	0.201	0.0718	0.564	0.002	−1.603
	Inverse variance weighted	0.281	0.068	1.151	0.078	−1.271

**Table 2:** Sensitivity analysis of Mendelian randomisation of physical activity, diet, and sedentary behaviour on atrial fibrillation.

	Heterogeneity Test						Pleiotropy Test		
	MR-Egger			Inverse Variance Weighted			MR-Egger		
	Q	Q_*df*	Q_pval	Q	Q_*df*	Q_pval	Intercept	se	*p*
Physical Activity	66.088	12	1.71 × 10^−9^	66.342	13	3.76 × 10^−9^	0.0079	0.037	0.834
Sedentary Behaviour	43.105	3	2.34 × 10^−9^	43.839	4	6.93 × 10^−9^	0.027	0.121	0.836
Diet	60.863	9	9.14 × 10^−10^	61.287	10	2.07 × 10^−9^	0.007	0.0295	0.808

**Table 3:** Mendelian randomised effects of physical activity, diet, and sedentary behaviour on heart failure.

		Heart Failure				
		or	or_lci95	or_uci95	*p*-Value	Beta
Physical Activity	MR-Egger	0.487	0.001	379.173	0.836	−0.720
	Weighted median	0.712	0.204	2.489	0.595	−0.339
	Inverse variance weighted	0.494	0.202	1.210	0.123	−0.705
Sedentary behaviour	MR-Egger	0.005	3.22 × 10^−5^	0.689	0.126	−5.358
	Weighted median	0.935	0.554	1.579	0.801	−0.067
	Inverse variance weighted	1.152	0.589	2.253	0.679	0.142
Diet	MR-Egger	0.0430	9.05 × 10^−5^	20.230	0.342	−3.151
	Weighted median	0.877	0.253	3.036	0.836	−0.131
	Inverse variance weighted	0.270	0.0740	0.990	0.048	−1.310

**Table 4:** Sensitivity analysis of Mendelian randomised effects of physical activity, diet, and sedentary behaviour on heart failure.

	Heterogeneity Test						Pleiotropy Test		
	MR-Egger			Inverse Variance Weighted			MR-Egger		
	Q	Q_*df*	Q_pval	Q	Q_*df*	Q_pval	Intercept	se	*p*
Physical Activity	11.335	12	0.500	11.335	13	0.583	0.0001	0.025	0.996
Sedentary Behaviour	5.921	3	0.116	15.234	4	0.004	0.158	0.073	0.118
Diet	19.288	9	0.023	20.061	10	0.029	0.016	0.026	0.563

## Data Availability

The data used in this study are publicly available from the UK Biobank (https://www.ukbiobank.ac.uk) and the FinnGen Project (https://www.finngen.fi/en). Access requires formal application and compliance with the respective data usage policies.
